# Individual courses of low back pain in adult Danes: a cohort study with 4-year and 8-year follow-up

**DOI:** 10.1186/s12891-016-1377-0

**Published:** 2017-01-21

**Authors:** Per Kjaer, Lars Korsholm, Charlotte Leboeuf-Yde, Lise Hestbaek, Tom Bendix

**Affiliations:** 1 Department of Sports Science and Clinical Biomechanics, University of Southern Denmark, Campusvej 55, DK-5230 Odense M, Denmark; 2 Research Department, Spine Centre of Southern Denmark, Hospital Lillebaelt, Department of Regional Health Research, University of Southern Denmark, Odense M, Denmark; 3 Center for Rheumatology and Spine Diseases, Rigshospitalet, University of Copenhagen, Copenhagen, Denmark; 4 Novo Nordisk, Bagsværd, Denmark; 5 Nordic Institute of Chiropractic and Clinical Biomechanics, Odense M, Denmark

**Keywords:** Low back pain, Epidemiology, Trajectories, Risk, Course

## Abstract

**Background:**

Few longitudinal studies have described the variation in LBP and its impact over time at an individual level. The aims of this study were to: 1) determine the prevalence of LBP in three surveys over a 9-year period in the Danish general population, using five different definitions of LBP, 2) study their individual long-term courses, and 3) determine the odds of reporting subsequent LBP when having reported previous LBP.

**Methods:**

A cohort of 625 men and women aged 40 was sampled from the general population. Questions about LBP were asked at ages 41, 45 and 49, enabling individual courses to be tracked across five different definitions of LBP. Results were reported as percentages and the prognostic influence on future LBP was reported as odds ratios (OR).

**Results:**

Questionnaires were completed by 412 (66%), 348 (56%) and 293 (47%) persons respectively at each survey. Of these, 293 (47%) completed all three surveys. The prevalence of LBP did not change significantly over time for any LBP past year: 69, 68, 70%; any LBP past month: 42, 48, 41%; >30 days LBP past year: 25, 27, 24%; seeking care for LBP past year: 28, 30, 36%; and non-trivial LBP, i.e. LBP >30 days past year including consequences: 18, 20, 20%. For LBP past year, 2/3 remained in this category, whereas four out of ten remained over the three time-points for the other definitions of LBP. Reporting LBP defined in any of these ways significantly increased the odds for the same type of LBP 4 years later. For those with the same definition of LBP at both 41 and 45 years, the risk of also reporting the same at 49 years was even higher, regardless of definition, and most strongly for seeking care and non-trivial LBP (OR 17.6 and 18.4) but less than 11% were in these groups.

**Conclusion:**

The prevalence rates of LBP, when defined in a number of ways, were constant over time at a group level, but did not necessarily involve the same individuals. Reporting more severe LBP indicated a higher risk of also reporting future LBP but less than 11% were in these categories at each survey.

## Background

Low back pain (LBP) is now rated as one of the most common [1], costly and disabling health conditions worldwide [2]. To date, the search for a cure has not been successful. One difficulty in the evaluation of the efficacy of treatments is that the natural course of back pain is not well understood. Another difficulty is that the identification of relevant subgroups for targeted treatment, prevention and care is still a challenge [3]. There is a view that higher priority should be given to identifying people at risk of developing chronic or recurrent disabling LBP in order to differentiate these from people with more benign LBP conditions [4]. Understanding different course trajectories of LBP may be helpful in this process.

Many studies have addressed the prevalence of LBP throughout the world and these have been summarised in reviews [1, 5–7]. The definitions of LBP and their prevalence estimates varied considerably with the heterogeneity of studies, making interpretation of the extent and, particularly, the impact, of LBP difficult. Nevertheless, chronic LBP conditions (usually defined as lasting for more than 3 months) with consequences have been reported in 6–20% of the adult population [8, 9].

The course of LBP is highly variable with LBP occurring in transient, recurrent or chronic phases [10]. However, longitudinal studies that describe individual courses of LBP are not very common. A recent systematic review included eight studies [11], where the authors found that among those reporting LBP at baseline, between 38 and 88% still reported LBP at follow up at various intervals. Another review of globally reported LBP estimated recurrence rates within one year to vary between 24 and 80% [5]. However, both reviews revealed a large variation in definitions of LBP, age ranges, occupational groups, time to, and number of follow ups, which makes it difficult to draw conclusions about the individual courses of LBP.

A recent paper reported on the stability in reporting ‘days with LBP’ in three categories (defined as no days, 1–30 days and >30 days with pain in the past year) and found that the prevalence in the population was relatively stable over 8 years and that approximately half of the individuals reported the same number of days at the subsequent follow up [12]. Furthermore, it was shown that people shifted the reporting mainly to the neighbouring category, not two categories away. In this paper, we will report on a particular cohort of people, but use other definitions of LBP. Previous studies have used different definitions of LBP, but usually not several per study. Therefore, we do not know how reporting patterns of LBP change with its definition.

Our intentions were to provide a deeper understanding of the nature of reporting LBP over time, and hopefully, to add to the process of identifying people with unfavourable prognoses, who may need secondary prevention of, and care for, LBP. The aims of this study were to: 1) determine the prevalence of LBP in three surveys over a 9-year period in the general population, using five different definitions of presumable increasing severity, 2) study their individual long-term courses, and 3) determine the odds of reporting subsequent LBP when having reported previous LBP.

## Methods

### Study participants and sampling

The Office of Civil Registrations identified a sample of 40-year-old Danes in the Year 2000 from the general population. Every ninth 40-year-old person from the approximately 500,000 inhabitants in the county of Funen, Denmark, born in Denmark, was selected and invited to participate in the study ‘Backs on Funen, Denmark’, a longitudinal study investigating LBP and its potential risk factors. The study protocol has been described in detail elsewhere [13].

### Study procedures

At the baseline visit (mean age 41, range 40–41), participants completed questionnaires about their LBP, had a lumbar MRI scan and a clinical examination of their lumbar spine. At 45 and 49 years of age, the participants were re-invited to have an MRI scan. Again, they were asked to complete the same questionnaires about their LBP.

### LBP variables and validity

The questionnaires contained questions that have previously been used in Scandinavian studies and have been partly validated [14–16]. All data were manually entered into Epidata [17]. From the baseline questionnaires, data were checked for consistency and logical errors, as reported elsewhere [18]. At age 45, all data were entered twice using the same tool, and edit checks were performed. At age 49, most of the participants completed an electronic questionnaire using the software, SurveyXact [19]. A small proportion of the participants completed paper questionnaires (69/293 = 23%), from which answers were entered twice into SurveyXact to validate data entry.

Five LBP definitions were used with the intent that these would reflect gradually increasing severity:

1. LBP within the past year defined by replying “Yes” to the question: “Have you had trouble with the lowest part of your back (picture provided) during the past year?” (hereafter referred to as ‘year’)
2. LBP within the past month defined by replying “Yes” to the question: “Have you had trouble with the lowest part of your back during the past month?” (‘month’)
3. LBP for more than 30 days within the past year by replying “Yes” to the question: “Have you had trouble with the lowest part of your back for more than 30 days within the past year?” (‘>30 days’)
4. Seeking care for LBP within the past year defined by replying “Yes” to the question: “Have you sought care during the past year due to trouble with the lowest part of your back? And if so, from whom?” by indicating one or more of the following: general practitioner, emergency service, specialist, out-patient clinic, hospitalised, chiropractor, physiotherapist, other treatment” (‘seeking care’)
5. LBP for more than 30 days with consequences defined by replying “Yes” to the question: “Have you had trouble with the lowest part of your back for more than 30 days during the last year and with at least one consequence of seeking care, reduced time at work, changed work function or reduction in leisure time activities?” (‘non-trivial’)

**Fig. 1:**
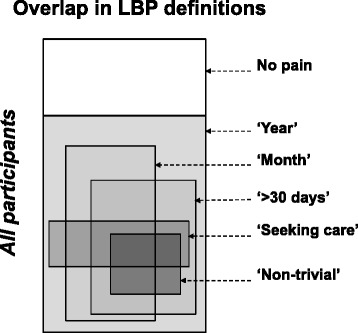
Overlap in Low Back Pain (LBP) definitions. The white area indicates those without LBP the past year. LBP within the past ‘Year’ includes all other definitions. LBP within the past ‘month’ includes many of the remaining definitions, but not all. ‘>30 days’ includes all ‘non-trivial’, but not all ‘seeking care’. ‘Seeking care’ for LBP within the past year can co-exist with any definition. The sizes of the areas do not indicate the proportion of individuals within each definition

The definitions are not mutually exclusive and an individual could belong to more than one of these categories (see Fig. 1).

### Statistical methods

Drop-out rates in those reporting and not reporting LBP within each category of LBP were calculated as percentages with 95% confidence intervals (CIs). CIs were compared in order to identify statistically significant differences in drop-out rates by LBP status.

The prevalence estimates of LBP were reported for the five definitions of LBP for the three surveys with all responders included. To test for statistically significant differences in prevalence rates at the three measurement time points, a test for trend, accounting for repeated measures, was performed.

The different patterns of reporting LBP over time per individual were graphed and the percentages of individuals entering and staying in each of the LBP categories at both follow-up periods were reported. Furthermore, the consistency in reporting within the same LBP definitions from age 41 to 49 was assessed by grouping the three possibilities into ‘each time’, ‘sometimes’ or ‘never’. The proportion of people within each of these groups was reported. For the longitudinal part of the study, we exclusively used data from those participants who completed the questionnaires at all three surveys.

LBP at the preceding survey as a predictor of subsequent LBP was expressed as odds ratios (OR) obtained from logistic regression (both from age 41 to 45 and from age 45 to 49).

Patterns of LBP at ages 41 and 45 (yes-yes, yes-no, no-yes, no-no) as risk factors of LBP at age 49 were studied. From logistic regression, ORs were estimated for each combination with the ‘no-no’ group as the reference. Further to this, the probability of belonging to a certain combination and the corresponding probability ratio to the no-no group were calculated. A test for interaction between LBP at age 41 and LBP at age 45 was performed using logistic regression within each of the five LBP definitions.

### Ethics

The study was approved by the The Regional Committees on Health Research Ethics for Southern Denmark (ref. no. 20000042) and by the Danish Data Protection Agency (ref. no. 2000-53-0037). All participants signed a written consent form.

## Results

### Participation

At baseline, 412 (66%) people out of the invited 625 participated at the age of 41 years, 348 (56%) participated again at 45 years, and 293 (47%) at 49 years. A total of 293 (47%) participated at all three time points. Details about the sex of the participants are shown in Table 1.

**Table 1:** Proportions of participants in the various LBP definitions

		41 year *n* = 412	45 years *n* = 348	49 years *n* = 293
Sex	male n (%)	198 (48.1%)	159 (46.1%)	136 (46.4%)
Low back pain definitions % [95% confidence interval]	past year	69%[64;73]	68%[63;73]	70%[64;75]
	past month	42%[38;47]	48%[43;54]	41%[35;46]
	>30 days/year	25%[21;29]	26%[21;30]	24%[19;28]
	seeking care*	*28%[23;32]*	*29%[24;34]*	*36%[30;41]*
	non-trivial	18%[15;22]	20%[16;24]	20%[15;24]

*p = 0.0044 (test for trend)

### Drop-out analyses

In general, most of the people who dropped out belonged to the groups previously reporting LBP (see Table 2). However, no statistically significant differences in proportions were found between participants with and without LBP. More men than women dropped out (19 and 31% versus 13 and 27% at the ages 45 and 49, respectively) but the differences were not statistically significant.

**Table 2:** Drop-out analyses

LBP	Reported previously	Age 45		Age 49	
		%	[95% CI]	%	[95% CI]
‘year’	no	11.7	[6.1;17.4]	19.1	[11.6;26.6]
	yes	17.3	[12.8;21.7]	14.3	[9.8;18.8]
‘month’	no	14.4	[9.9;18.9]	14.0	[8.8;19.1]
	yes	17.1	[11.5;22.8]	17.8	[11.9;23.6]
‘>30 days’	no	13.9	[10.0;17.4]	14.0	[9.7;18.2]
	yes	20.6	[12.6;28.6]	21.1	[12.5;29.7]
‘seeking care’	no	14.4	[10.4;18.4]	14.1	[9.8;18.5]
	yes	18.4	[11.2;25.7]	20.0	[12.0;28.0]
‘non-trivial’	no	14.6	[10.8;18.4]	14.4	[10.2;18.5]
	yes	19.7	[10.6;28.9]	21.4	[11.6;31.3]
sex	male	18.7	[13.2;24.2]	31.3	[24.8;37.8]
	female	12.6	[8.1;17.1]	26.6	[20,7;32.6]

The percentages of people dropping out at the age of 45 and 49 by LBP definition at the previous survey and by sex

### Prevalence of LBP

As shown in Table 1, the prevalence rates within each of the five different definitions of LBP were stable over time. Only ‘seeking care’ showed a statistically significant increase from 45 to 49 years. In general, the prevalence estimates decreased with the severity of the LBP definition.

### Variability in LBP reporting

More than half of those who had reported LBP within a certain definition did the same at the next survey (Table 3). Approximately four out of ten reported LBP within the same definitions at all three time points except for ‘year’, where it was two-thirds.

**Table 3:** Four-year patterns of reporting LBP

	41 → 45 years				45 → 49 years			
	Percentage of individuals reporting:		Odds of reporting recurrent LBP		Percentage of individuals reporting:		Odds of reporting recurrent LBP	
Category LBP	new at 45	recurrent	OR	[95% CI]	new at 49	recurrent	OR	[95% CI]
‘none’	23%	50%	3.8	[2.2;6.4]	21%	53%	4.3	[2.5;7.4]
‘year’	50%	77%	3.5	[2.2; 5.6]	47%	79%	4.3	[2.5; 7.4]
‘month’	37%	65%	3.1	[2.0; 4.9]	29%	54%	2.9	[1.8; 4.8]
‘>30 days’	15%	63%	9.9	[5.6; 17.5]	15%	51%	5.9	[3.2; 10.7]
‘seeking care’	20%	52%	4.2	[2.5; 6.9]	25%	64%	5.2	[3.0; 9.0]
‘non-trivial’	13%	56%	8.8	[4.7; 16.2]	12%	53%	8	[4.2; 15.5]

Overview of individuals reporting new or recurrent LBP after 4 years and odds ratios (OR) for recurrence with 95% confidence intervals (CI) as a measure of positive LBP relative to negative LBP. *N* = 293 participating at all three time points

**Fig. 2:**
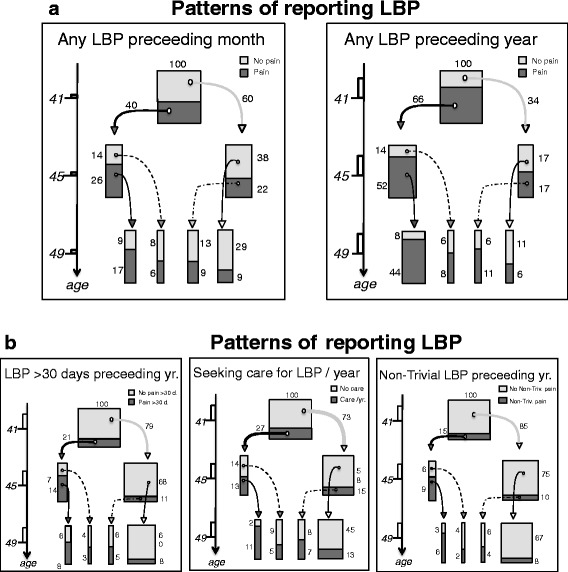
**a** Patterns of reporting LBP. Courses of the two least severe of the five definitions of Low Back Pain (LBP) across the three surveys. Each diagram is normalized to 100 individuals starting from our 293 participants tested at all three time points. The small boxes to the left illustrate the periods the data were sampled. The relative sizes of the various fractions are depicted by the width of the columns. **b** Patterns of reporting LBP. Courses of the three most severe definitions of Low Back Pain (LBP) across the three surveys. Each diagram is normalized to 100 individuals starting from our 293 participants tested at all three time points. The small boxes to the left illustrate the periods the data were sampled. The relative sizes of the various fractions are depicted by the width of the columns. ‘Non-trivial LBP’ means pain >30 days + seeking care + reduced functional level at work and/or home

The tracked patterns of the different definitions of LBP reporting are shown in Fig. 2a and b.

During the entire study period, 11% never reported any type of LBP, 45% reported some type of LBP at one or two of the three surveys, and 44% reported having some type of LBP at each time point. Less than 11% reported impactful LBP across the three surveys (Table 4).

**Table 4:** Long-term patterns of reporting LBP

	Consistency in LBP categories at the ages 41–45–49		
LBP	Each time	Sometimes	Never
none	11%	45%	44%
‘year’	44%	45%	11%
‘month’	17%	54%	29%
‘>30 days’	8%	32%	60%
‘seeking care’	11%	44%	45%
‘non-trivial’	6%	27%	67%

The percentage of individuals reporting LBP in various patterns: ‘*Each time’* refers to the percentage of people reporting a specific category of LBP at all time points; ‘*Sometimes’:* the percentage of people with changing status over time; ‘*Never’:* those people never reporting this type of LBP (*N* = 293 participating at all three time points)

### LBP as a risk for future LBP

The odds of reporting the same definition of LBP after 4 years were between 2.9 and 9.9, most markedly for those reporting LBP in the definitions ‘>30 days’ (OR 9.9) and ‘non-trivial’ (OR 8.0) (See Table 3).

As shown in Table 5, all definitions of LBP were associated with significantly increased odds of reporting the same type of LBP at age 49. A pattern of reporting LBP at both previous time points (yes-yes) increased the odds most markedly at the third survey, whereas reporting in a yes-no or no-yes fashion showed more moderate ORs. ‘Seeking care’ and ‘non-trivial’ were associated with markedly higher odds for the yes-yes combination, (OR 17.6 and 18.4, respectively) than the other definitions of LBP (OR between approximately 6 and 10). The probability ratios indicate the same risk patterns except ‘>30 days/year’ carry a higher risk of future LBP than ‘Seeking care’ when calculated this way. No interactions were identified.

**Table 5:** Patterns of reporting LBP at 41 and 45 as risks of LBP at 49

LBP	History		LBP at 49 years of age	Probability of LBP at 49 given ‘history’	Probability ratio with the no-no group as reference
	LBP 41	LBP 45	OR [95% CI]	%	
‘year’	yes	yes	8.9 [4.3;18.3]	84	2.28
	no	yes	3.3 [1.5;7.6]	66	1.80
	yes	no	2.6 [1.1;6.1]	60	1.63
	no	no	1.0	37	1.00
‘month’	yes	yes	5.9 [3.1;11.2]	64	2.76
	no	yes	2.4 [1.2; 4.7]	42	1.82
	yes	no	2.5 [1.2;5.3]	43	1.85
	no	no	1 .0	23	1.00
‘>30 days/year’	yes	yes	9.5 [4.5;20.2]	58	4.60
	no	yes	5.1 [2.2;11.6]	42	3.35
	yes	no	4.0 [1.5;10.5]	36	2.91
	no	no	1.0	13	1.00
‘seeking care’	yes	yes	17.6 [6.9;45.4]	84	3.70
	no	yes	3.0 [1.5;6.0]	47	2.05
	yes	no	2.0 [0 .9;4.1]	37	1.61
	no	no	1 .0	23	1.00
‘non-trivial’	yes	yes	18.4 [7.2;46.9]	69	6.35
	no	yes	5.0 [2.1;11.8]	38	3.48
	yes	no	3.1 [1.0; 9.6]	28	2.55
	no	no	1.0	11	1.00

Patterns of LBP at the ages of 41 and 45 (columns 2 and 3) as risk factors of LBP at age 49 (column 4). OR = odds ratios with 95% confidence intervals (CI), the probability of being in the subgroup as percentage (%) and the probability ratio with the no-no group

## Discussion

### Main results

To our knowledge, this is the first study that reports longitudinal data from several definitions of self-reported LBP with varying impact in a cohort of the same age from the general population. Regardless of the definition of LBP, the main findings were that: (i) the proportions of people reporting each specific definition of LBP were constant over time, although fluctuations occurred for most individuals; (ii) the proportion of people reporting long-lasting, care-seeking and non-trivial LBP at all three surveys was relatively small; (iii) those belonging to the most ‘severe’ LBP categories had higher risk of reporting it again in the subsequent surveys.

### Definitions of LBP

In this study, we chose to include five different definitions of LBP, largely reflecting five severity or impact levels, with the final level based on a combination of questions that were adapted from previously used questionnaires in epidemiologic studies [14–16]. Internationally, attempts have been made to create uniform definitions of LBP [20] and LBP episodes [21] but so far these definitions have not been fully implemented in research. It still remains to be evaluated whether the suggested definitions of LBP and episodes are meaningful in a clinical setting, as well as in epidemiology, as discussed in previous papers using SMS to describe the course of LBP more closely [22–25].

We combined ‘>30 days’ and ‘seeking care’ with limitations of activity and participation into the variable ‘non-trivial’ because we believed that this would reflect our most serious LBP definition. This sub-group of people consumes a substantial proportion of society’s health resources, and there has been a recent suggestion that it would be ideal to routinely include pain, activity limitations and social factors in the evaluation of clinical and research outcomes for patients with LBP [26].

### Comparisons with other studies

Our prevalence estimates were similar to findings reported by others for the LBP definitions ‘seeking care’ and ‘non-trivial’ [8] but somewhat higher for LBP ‘month’ and ‘year’ [1, 5–7, 27]. However, the heterogeneous data collection methods and definitions of LBP severity used in these studies, including variation in frequency and timing of follow-up, as well as differences in study samples and age groups, make direct comparisons difficult. Our higher prevalence rates may reflect that people who had LBP were more likely to accept participation in our time-demanding study with a one-hour MRI scan plus additional testing which could have explained our somewhat higher estimates for LBP ‘month’ and ‘year’. Nevertheless, the response rate at follow-up was higher among those without LBP at baseline.

In our study, almost half of the participants reported a fluctuating pattern of LBP across the different definitions, but rarely in the direction of no pain, thus confirming that LBP is a recurrent condition [28]. The literature from long-term cohort studies is sparse, but generally in line with our findings [8, 9, 29–31]. Van Oostrom et al. have reported similar results with approximately 30% reporting longstanding LBP in a fluctuating pattern, of which only 6% consistently reported LBP at three time points over a 10-year period, which compares well with the prevalence of reporting ‘non-trivial’ LBP at all three surveys [8].

Waxman et al.’s community-based study included 1,455 individuals, one-third of whom reported persistent LBP in two surveys with a three-year interval [9]. Half of these reported acute LBP at the next or previous survey. Cassidy et al. followed a cohort of 1,110 people from the general population with two follow-ups over one year [29]. In those people with LBP, less than one-third resolved within a year, one fifth had recurrences, and less than 1% developed severe and disabling LBP, which is a smaller proportion than we found. In another study of 252 people, Cedrashi et al. found the population fraction diagnosed as chronic to be reasonably stable over a three-year period [30] with about half of the individuals labelled as chronic at baseline being chronic also at follow-up. In Hestbaek et al.’s study with a five-year follow-up, LBP fluctuated with periodic attacks and temporary remissions, and also while long-lasting LBP (>30 days per year) was reported by one quarter, it was repeatedly reported by only about 10% [31].

Previous LBP has been suggested as one of the strongest predictors or prognostic factors of future LBP [32, 33]. In our study, all definitions of LBP indicated a risk of future reporting of LBP within the same definition. However, people reporting ‘non-trivial’ LBP had the highest odds of LBP after 4 years (OR >8) and, if reporting it at 41 and 45 years, there was a very high risk of reporting it again at 49 years (OR > 18). It is noteworthy that the embedded variables ‘seeking-care’ and ‘>30 days’ had similar patterns of risk throughout our study.

### Strengths and weaknesses of the study

It is a strength of our study that it was conducted using a representative sample from the general population with only a slight over-representation of people with higher education [18], that the same people were followed over 8 years and that they were all of the same age. The same questionnaires were used at all three measurement time points, the follow-up rate was reasonable and people dropping out of the study were not markedly different from those who stayed in it. Those, who stayed in, were less likely to have LBP for example. In previous reports from the same cohort, we have thoroughly analysed dropouts and compared a number of psychological, social and biological factors [12, 34]. We found that people dropping out compared with those who remained in the study were somewhat more likely to be retired, to have a lower level of education, and to have types of LBP with more impact, but none of these factors were statistically significant [12]. Furthermore, we transparently reported the response rate as a percentage of the invited people, which is often lacking in epidemiologic studies [11].

The study sample may be biased towards a population with LBP because participants were offered a thorough examination of the lumbar spine including MRI. By the end of the study, less than half of the sampled people participated, but this was found not to be associated with the baseline characteristics as shown in the dropout analysis. In summary, we therefore believe the study sample to be fairly representative of the general middle-aged Danish population and thus the estimates of risks of future LBP to be valid.

### Implications

#### Clinical implications

The fact that several individual courses exist and that an episode with LBP will often resolve is a highly positive message to the person presenting in the clinic with LBP. On the other hand, reporting of a previous LBP episode, or even worse, several previous episodes, and in particular LBP with consequences, significantly increases the risk of future LBP episodes. This knowledge is helpful for both patient and clinician, by introducing a realistic insight into the prognosis.

#### Research implications

In this study, it was evident that when applying the more ‘severe’ definitions of LBP (‘>30 days’, ‘seeking care’, and ‘non-trivial’) as risk factors for future LBP of the same definitions, the associations were stronger than for ‘year’ and ‘month’. We therefore suggest that composite measures of LBP outcomes should be further explored in future epidemiologic studies of risk factors and less attention should be paid to the LBP definitions ‘year’ and ‘month’, which may include both slight LBP with low clinical impact and severe disabling LBP.

When trying to understand the course of LBP, a long timespan between surveys will disguise the fluctuations in LBP in the intervening period. This has to be investigated more closely. Having a fluctuating outcome complicates the investigation of risk factors for future LBP. We therefore encourage researchers to further study the causes of fluctuating LBP. This may offer information about underlying factors that accelerate or inhibit recurrence, and as such, may provide a more accurate prognosis for a specific person, although one should always keep in mind that general epidemiology cannot be translated to specific estimates for specific individuals.

## Conclusion

This study confirmed that in a population-based sample of middle-aged people, LBP is a common, changeable condition that increases the odds of future LBP. Three surveys undertaken at the ages of 41, 45 and 49 showed almost identical prevalence rates for each definition of LBP, with the less severe definitions being most common at the population level. However, at an individual level, LBP was reported differently at the three surveys with about half reporting the same LBP severity at the next survey.

This study also showed that people with the most severe definitions of LBP had a much higher risk of also reporting future LBP. This group of people presents as a subgroup that has a less favourable prognosis, and who may need secondary prevention and care if, indeed, this is possible. However, future research should investigate the relevance of new definitions, which include patterns of reporting, duration, activity and participation limitations.

## Abbreviations

CI: Confidence intervals
LBP: Low back pain
MRI: Magnetic resonance imaging
OR: Odds ratio

## References

[1] Hoy D, Bain C, Williams G, March L, Brooks P, Blyth F, Woolf A, Vos T, Buchbinder R (2012). A systematic review of the global prevalence of low back pain. Arthritis Rheum.

[2] Maniadakis N, Gray A (2000). The economic burden of back pain in the UK. Pain.

[3] Costa Lda C, Koes BW, Pransky G, Borkan J, Maher CG, Smeets RJ (2013). Primary care research priorities in low back pain: an update. Spine (Phila Pa 1976).

[4] Foster NE, Hill JC, O’Sullivan P, Hancock M (2013). Stratified models of care. Best Pract Res Clin Rheumatol.

[5] Hoy D, Brooks P, Blyth F, Buchbinder R (2010). The epidemiology of low back pain. Best Pract Res Clin Rheumatol.

[6] Volinn E (1997). The epidemiology of low back pain in the rest of the world. A review of surveys in low- and middle-income countries. Spine (Phila Pa 1976).

[7] Leboeuf-Yde C, Klougart N, Lauritzen T (1996). How common is low back pain in the Nordic population? data from a recent study on a middle-aged general Danish population and four surveys previously conducted in the Nordic countries. Spine.

[8] van Oostrom SH, Monique Verschuren WM, de Vet HC, Picavet HS (2011). Ten year course of low back pain in an adult population-based cohort--the doetinchem cohort study. Eur J Pain.

[9] Waxman R, Tennant A, Helliwell P (2000). A prospective follow-up study of low back pain in the community. Spine (Phila Pa 1976).

[10] Von Korff M (1994). Studying the natural history of back pain. Spine (Phila Pa 1976).

[11] Lemeunier N, Leboeuf-Yde C, Gagey O (2012). The natural course of low back pain: a systematic critical literature review. Chiropr Man Ther.

[12] Lemeunier N, Leboeuf-Yde C, Kjaer P, Gagey O (2013). Stability of low back pain reporting over 8 years in a general population aged 40/41 years at base-line: data from three consecutive cross-sectional surveys. BMC Musculoskelet Disord.

[13] Kjaer P, Leboeuf-Yde C, Korsholm L, Sorensen JS, Bendix T (2005). Magnetic resonance imaging and low back pain in adults: a diagnostic imaging study of 40-year-old men and women. Spine (Phila Pa 1976).

[14] Kuorinka I, Jonsson B, Kilbom A, Vinterberg H, Biering-Sorensen F, Andersson G, Jorgensen K (1987). Standardised Nordic questionnaires for the analysis of musculoskeletal symptoms. Appl Ergon.

[15] Biering-Sorensen F, Hilden J (1984). Reproducibility of the history of low-back trouble. Spine (Phila Pa 1976).

[16] Leboeuf-Yde C, Kyvik KO (1998). At what age does low back pain become a common problem? a study of 29,424 individuals aged 12–41 years. Spine (Phila Pa 1976).

[17] Bruus M. EpiData Data Entry, Data Management and basic Statistical Analysis System. In*.* Edited by Lauridsen JM, 3.1 edn. Odense Denmark: EpiData Association; 2000–2008.

[18] Kjaer P. Low back pain in relation to lumbar spine abnormalities as identified by magnetic resonance imaging. Dissertation. Ph.D. Odense: Dissertation, Faculty of Health Sciences - University of Southern Denmark; 2004.

[19] Rambøll_Management_Consulting (2008). SurveyXact. Aarhus, Denmark, Rambøll Group.

[20] Dionne CE, Dunn KM, Croft PR, Nachemson AL, Buchbinder R, Walker BF, Wyatt M, Cassidy JD, Rossignol M, Leboeuf-Yde C (2008). A consensus approach toward the standardization of back pain definitions for use in prevalence studies. Spine (Phila Pa 1976).

[21] de Vet HC, Heymans MW, Dunn KM, Pope DP, van der Beek AJ, Macfarlane GJ, Bouter LM, Croft PR (2002). Episodes of low back pain: a proposal for uniform definitions to be used in research. Spine.

[22] Leboeuf-Yde C, Lemeunier N, Wedderkopp N, Kjaer P (2013). Evidence-based classification of low back pain in the general population: one-year data collected with SMS Track. Chiropr Man Ther.

[23] Leboeuf-Yde C, Lemeunier N, Wedderkopp N, Kjaer P (2014). Absence of low back pain in the general population followed fortnightly over one year with automated text messages. Chiropr Man Ther.

[24] Kongsted A, Kent P, Axen I, Downie AS, Dunn KM (2016). What have we learned from ten years of trajectory research in low back pain?. BMC Musculoskelet Disord.

[25] Kongsted A, Kent P, Hestbaek L, Vach W (2015). Patients with low back pain had distinct clinical course patterns that were typically neither complete recovery nor constant pain. A latent class analysis of longitudinal data. Spine J.

[26] Deyo RA, Dworkin SF, Amtmann D, Andersson G, Borenstein D, Carragee E, Carrino J, Chou R, Cook K, DeLitto A (2014). Report of the NIH task force on research standards for chronic low back pain. Spine (Phila Pa 1976).

[27] Leboeuf-Yde C, Nielsen J, Kyvik KO, Fejer R, Hartvigsen J (2009). Pain in the lumbar, thoracic or cervical regions: do age and gender matter? a population-based study of 34,902 Danish twins 20–71 years of age. BMC Musculoskelet Disord.

[28] Stanton TR, Latimer J, Maher CG, Hancock M (2009). Definitions of recurrence of an episode of low back pain: a systematic review. Spine (Phila Pa 1976).

[29] Cassidy JD, Cote P, Carroll LJ, Kristman V (2005). Incidence and course of low back pain episodes in the general population. Spine (Phila Pa 1976).

[30] Cedraschi C, Robert J, Goerg D, Perrin E, Fischer W, Vischer TL (1999). Is chronic non-specific low back pain chronic? definitions of a problem and problems of a definition. Br J Gen Pract.

[31] Hestbaek L, Leboeuf-Yde C, Engberg M, Lauritzen T, Bruun NH, Manniche C (2003). The course of low back pain in a general population. Results from a 5-year prospective study. J Manipulative Physiol Ther.

[32] Hestbaek L, Leboeuf-Yde C, Kyvik KO (2006). Is comorbidity in adolescence a predictor for adult low back pain? a prospective study of a young population. BMC Musculoskelet Disord.

[33] Dunn KM, Hestbaek L, Cassidy JD (2013). Low back pain across the life course. Best Pract Res Clin Rheumatol.

[34] Jensen TS, Bendix T, Sorensen JS, Manniche C, Korsholm L, Kjaer P (2009). Characteristics and natural course of vertebral endplate signal (Modic) changes in the Danish general population. BMC Musculoskelet Disord.

